# A New, Potent, and Placenta-Permeable Macrolide Antibiotic, Solithromycin, for the Prevention and Treatment of Bacterial Infections in Pregnancy

**DOI:** 10.3389/fimmu.2016.00111

**Published:** 2016-04-01

**Authors:** Jeffrey A. Keelan, Matthew S. Payne, Matthew W. Kemp, Demelza J. Ireland, John P. Newnham

**Affiliations:** ^1^King Edward Memorial Hospital, School of Women’s and Infants’ Health, University of Western Australia, Perth, WA, Australia

**Keywords:** macrolide antibiotics, intrauterine infection, prelabor rupture of membranes, *Ureaplasma*, *Mycoplasma*, pregnancy

## Abstract

Intrauterine infection–inflammation is a major cause of early preterm birth and subsequent neonatal mortality and acute or long-term morbidity. Antibiotics can be administered in pregnancy to prevent preterm birth either prophylactically to women at high risk for preterm delivery, or to women with diagnosed intrauterine infection, prelabor rupture of membranes, or in suspected preterm labor. The therapeutic goals of each of these scenarios are different, with different pharmacological considerations, although effective antimicrobial therapy is an essential requirement. An ideal antibiotic for these clinical indications would be (a) one that is easily administered and orally bioactive, (b) has a favorable adverse effect profile (devoid of reproductive toxicity or teratogenicity), (c) is effective against the wide range of microorganisms known to be commonly associated with intra-amniotic infection, (d) provides effective antimicrobial protection within both the fetal and amniotic compartments after maternal delivery, (e) has anti-inflammatory properties, and (f) is effective against antibiotic-resistant microorganisms. Here, we review the evidence from clinical, animal, and *ex vivo/in vitro* studies that demonstrate that a new macrolide-derived antibiotic – *solithromycin* – has all of these properties and, hence, may be an ideal antibiotic for the treatment and prevention of intrauterine infection-­related pregnancy complications. While this evidence is extremely encouraging, it is still preliminary. A number of key studies need to be completed before solithromycin’s true potential for use in pregnancy can be ascertained.

## Introduction

Preterm infants are at high risk of adverse outcomes, including both acute and long-term disability and death (1–4). Evidence from multiple clinical and animal studies suggests that the majority of early preterm deliveries (before 34 weeks’ gestation) arise as the result of intrauterine infection and inflammation (5, 6), although causation is hard to prove in any individual case. Ascending intrauterine infection occurs when bacteria residing in the vagina ascend and breach the cervical barrier, colonize and invade the fetal membranes and amniotic fluid (AF) – and sometimes infect the fetus itself (7, 8). When a vigorous inflammatory response ensues (typically manifested as histologic chorioamnionitis), this may trigger preterm labor and delivery (7, 9, 10). Microbial colonization of the amniotic cavity without a significant inflammatory response rarely manifests as a cause of preterm delivery (11–13).

In order to successfully prevent intra-amniotic infection-associated preterm birth and associated neonatal sequelae, an effective antibiotic therapy needs to be a core component of any pharmaceutical solution. Ideally an antibiotic administered antenatally for preterm birth prevention should (a) be easily administered and orally bioactive; (b) have a favorable adverse effect profile in pregnancy (devoid of reproductive toxicity or teratogenicity); (c) exhibit efficacy against the wide range of microorganisms known to be commonly associated with intra-amniotic infection; (d) be able to provide effective antimicrobial protection within both the fetal and amniotic compartments after maternal delivery; (e) possess anti-inflammatory properties; and (f) be effective against antibiotic-resistant microorganisms.

In this review, we discuss the key properties, benefits, and potential obstetric and perinatal applications of the novel antibiotic *solithromycin*. We suggest that solithromycin is the first antibiotic that may meet all of the above-mentioned criteria and as such has the potential to represent an exciting and major new advance in obstetric and perinatal medicine. We highlight the key indications where solithromycin may be of most benefit, and identify areas where further research is needed in order to facilitate the introduction of solithromycin into obstetric practice.

## Intrauterine Infection, Antibiotics, and Preterm Birth

**Figure 1:**
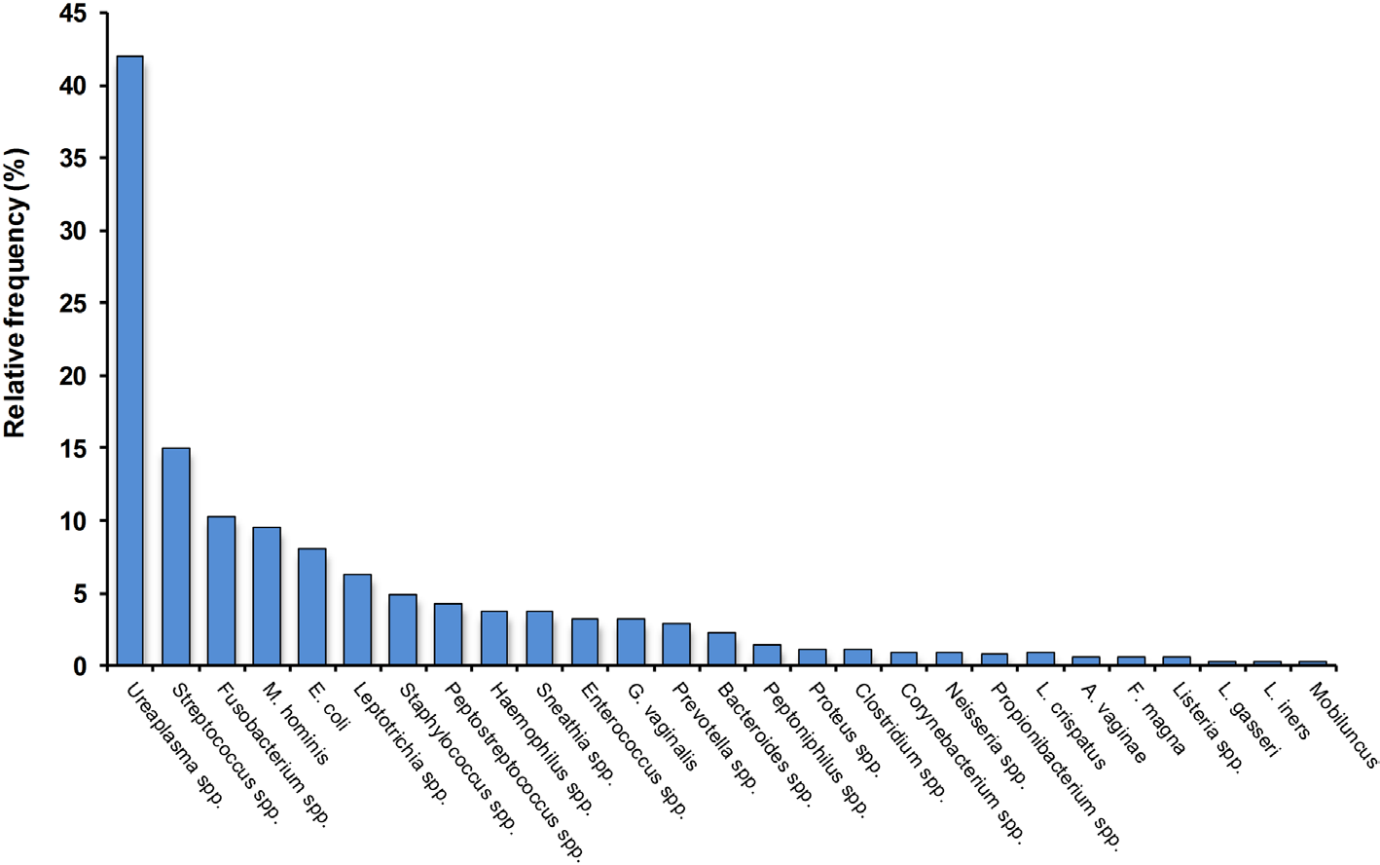
**Relative frequency of colonization by different bacteria of the amniotic cavity in preterm deliveries with intact membranes and intra-amniotic infection**. Note that more than one bacterium are frequently detected. Data, compiled from Ref. (16–19), are indicative only and will vary according to clinical and demographic characteristics, plus methodological differences.

Intrauterine infection and inflammation play a well-recognized role in the etiology of spontaneous preterm labor and birth, particularly in early preterm deliveries or those complicated by preterm prelabor rupture of membranes (PPROM) (6, 9, 12). A large number of microorganisms have been implicated in the etiology of preterm birth, including many organisms commonly found in normal vaginal microbiota as well as conditions associated with vaginal dysbiosis (Figure 1) (14–16). Some of the bacteria that commonly cause infection-driven preterm birth are common Gram-positive bacteria frequently found in the reproductive tract of pregnant women; some are more closely associated with oral microbes, while others are often found in women with abnormal vaginal microbiota [bacterial vaginosis (BV)] and/or are associated with reproductive tract infections (14, 17–19). In many preterm deliveries, multiple bacteria are present in the amniotic cavity (14, 17, 20). The incidence of confirmed intra-amniotic infection in preterm deliveries varies according to a variety of factors, including race and gestational age. In a recent analysis, Romero et al. reported that AF bacterial colonization rates are around 10–15% in preterm births overall, approaching 30% in extreme preterm births delivered before 30 weeks’ gestation (12). In an earlier review of the topic, DiGiulio described a frequency of AF infection ranging from 15 to 50%, with pregnancies complicated by PPROM having a similar infection rate (14). Intra-amniotic inflammation (with or without infection) is considerably more common, and increases more markedly with decreasing gestational age at delivery (12, 21, 22).

Bacteria of the class Mollicutes, in particular the “genital mycoplasmas,” such as *Ureaplasma parvum*, *Ureaplasma urealyticum*, and *Mycoplasma hominis*, are the most common group of ­microorganisms isolated from the amniotic cavity of preterm deliveries (9, 14). Vaginal colonization rates of these organisms in pregnant women ranges from 35 to 90% for *Ureaplasma* spp. and 5–75% for *Mycoplasma hominis* (23). Dual colonization with both microorganisms is approximately fourfold more common in women with preterm vs. term deliveries (23, 24). Most studies with a preterm birth endpoint have reported a significant association with intrauterine *Ureaplasma* sp. colonization and preterm birth (25); studies of AF and placental tissues obtained from preterm deliveries show a clear link between *Ureaplasma* colonization, a vigorous inflammatory response, and preterm delivery (24–29).

The clinical evidence is supported by experimental studies consistent with causality (30). Using a pregnant sheep model (31), we reported that intra-amniotic injection with *Ureaplasma parvum* resulted in chronic chorioamnionitis accompanied by pro-inflammatory cytokines in the AF and enhanced lung maturation. Experiments in Rhesus macaques have shown that intra-amniotic *Ureaplasma* sp. injection also drives intrauterine cytokine and prostaglandin production, preterm labor, and chorioamnionitis, replicating the disease pathogenesis and ontogeny observed in human pregnancy (32, 33). Together, these and other studies have shown that robust intrauterine inflammation sufficient to cause preterm birth can be induced by *Ureaplasma* sp. colonization of the amniotic cavity (25). However, it is important to note that around half of all preterm deliveries with intra-amniotic infection contain bacteria other than the genital Mycoplasmataceae, and a large number of bacterial species have been associated with inflammation-driven preterm birth (14, 17, 18, 34).

A number of clinical trials of maternal antibiotic administration have been performed to attempt to prevent or treat intrauterine infection with the aim of reducing the rates of preterm birth and associated neonatal morbidities. As discussed in detail in this series by Lamont (35), some recent meta-analyses have concluded that antibiotic treatment of BV does not prevent preterm birth or improve neonatal outcomes (36–41). Metronidazole and clindamycin are the two most studied antibiotics. It should be noted here that conventional treatment of BV results in relatively high recurrence rates (42–44), and that the antibiotics commonly used to treat BV show only weak activity against *Mycoplasma hominis* (erythromycin, azithromycin, metronidazole) or *Ureaplasma* spp. (metronidazole, clindamycin) (14). High concentrations of these antibiotics may be required for efficacy that may not be achievable with standard oral doses due to their comparatively low oral bioavailability or adverse effects profile.

However, there are some studies that suggest that prophylactic antibiotic administration can be effective – if given before 20 weeks’ gestation (35). This is presumably because antimicrobial therapy is most effective and beneficial when administered prior to colonization of the amniotic cavity (45, 46). A retrospective study of clindamycin treatment of women with genital mycoplasmas at high risk of preterm birth found a small but significant reduction in preterm birth rates and neonatal complications (47). In addition to clindamycin, azithromycin may also be effective. In non-human primates, Grigsby and colleagues showed that 10 days of high-dose maternal azithromycin treatment delays preterm labor induced by experimental intra-amniotic *Ureaplasma* spp. infection and prevents fetal inflammatory response (32). We recently showed in our ovine model that a 4-day course of azithromycin-delivered maternally (10 mg/kg i.v.) eradicated intra-amniotic *Ureaplasma parvum* infection (48). Surprisingly, there are only two clinical studies of macrolide treatment of vaginal *Ureaplasma* spp. colonization on pregnancy outcome, the results of which are inconclusive (49, 50).

In addition to difficulties surrounding diagnosis of infection and the appropriate selection of antibiotics, a fundamental reason for the lack of success of antibiotic trials for preterm birth prevention may lie in the limitations of the antibiotics employed. While macrolide antibiotics, such as erythromycin and azithromycin, are considered effective in treating important microorganisms, such as *Ureaplasma* spp., and are generally free of serious maternal and fetal side effects, their potency against genital mycoplasmas is not high, and there is growing prevalence of antibiotic resistance in these organisms (23). Studies have shown that maternal erythromycin administration is largely ineffective in eradicating intrauterine infection (39, 51, 52). This is likely due to poor transplacental passage of macrolides, estimated to be only 2–4% (53, 54). We previously showed in our pregnant sheep model that maternal macrolide administration fails to deliver effective levels of antibiotic to either the fetal circulation or the amniotic cavity (55) and does not eradicate intra-amniotic *Ureaplasma parvum* infection (52). Human studies confirm that the degree of maternal-to-fetal (M:F) passage of macrolides, such as erythromycin and azithromycin, is low and variable (53, 54), while the extent of maternal-to-amniotic transfer is only marginally greater. Antibiotics with better maternal-amniotic-fetal transfer properties and enhanced potencies against key bacterial pathogens are required to eliminate intra-amniotic infection and prevent significant neonatal morbidity and mortality.

## Solithromycin: Pharmacodynamics and Antimicrobial Properties

Solithromycin, a fourth-generation macrolide derived from clarithromycin, is a novel fluoroketolide antibiotic being developed by Cempra Inc. (Chapel Hill, NC, USA) for the treatment of community-acquired pneumonia and a variety of other indications (56, 57). It exhibits broad-spectrum activity against Gram-positive and some Gram-negative organisms, including many that are resistant to other macrolide antibiotics (58–67). It is acid stable (63) and has excellent oral bioavailability (~70%), superior to the approved macrolides (68–70). Solithromycin also demonstrates excellent tissue uptake and accumulation, important when considering its activity in tissues infected with intracellular pathogens, such as *Ureaplasma* sp. (70, 71).

Like other macrolides, solithromycin contains a 14-atom lactone ring structure and selectively binds to the peptide exit tunnel of the bacterial ribosome, blocking subunit assembly, and mRNA translation and protein synthesis (72). It has three key structural features that distinguish it from first- and second-generation macrolides: a keto group replacing the cladinose moiety (hence, the origin of the class name “ketolide”), a fluoro group at the C2 position of the lactone ring, and an aryl–aryl side chain at C11–C12. Deletion of the cladinose structure renders the molecule insensitive to methylation-dependent resistance. Hydrogen bonding via the amino-phenyl headgroup of the C11,C12 side chain is primarily responsible for solithromycin’s high-affinity bacterial ribosomal binding properties, while the fluoro group enhances binding in some macrolide-resistant strains. Ketolides are generally less susceptible than macrolides to bacterial efflux pumps, enhancing their efficacy in some species. In addition, there is some evidence that solithromycin selectively blocks translation of specific polypeptides, which confers additional antimicrobial efficacy over traditional macrolides (72).

Solithromycin has been shown to exhibit excellent activity against many of the microbial species known to be associated with infection-associated preterm delivery (Table 1), including *Ureaplasma* spp., *Mycoplasma* spp., Group B streptococci, staphylococci, and *Chlamydia trachomatis* (59, 61, 65–67, 73, 74). Indeed, although solithromycin has not yet been tested on all relevant microorganisms, from the established efficacy profile of its parent drug clarithromycin, it is likely that solithromycin will be effective against all bacteria known to be associated with intra-amniotic infection. We recently showed that its potency against *Ureaplasma* spp. is ~30 times greater than azithromycin *in vitro* (75). Importantly, no strains of *Ureaplasma* were resistant to solithromycin, and both *Ureaplasma parvum* and *Ureaplasma urealyticum* were susceptible, with overall MIC_90_ values 125 ng/ml (compared to 2000 ng/ml for azithromycin).

**Table 1:** **Comparison of antimicrobial efficacy (MIC_50_ and MIC_90_ values) of solithromycin vs. four other relevant antibiotics against a range of important bacteria**.

Organism (number of strains)	Solithromycin		Macrolides^a^		Levofloxacin		Penicillins^b^		Doxycyclin		Reference
			
	*MIC_50_*	*MIC_90_*	*MIC_50_*	*MIC_90_*	*MIC_50_*	*MIC_90_*	*MIC_50_*	*MIC_90_*	*MIC_50_*	*MIC_90_*	*#*
*Streptococcus pneumoniae (1363)*	<30	120	<250	>2,000^(Er)^	1,000	1,000	<30	2000^(P)^			(67)
*Streptococcus pyogenes (124)*	60	500	8,000	>64,000^(Az)^	500	1000	15	15^(AC)^			(76)
*Streptococcus agalactiae*^c^ *(GBS)^R^ (62)*	30	125	>8,000	>8,000^(Az)^							(59)
*Streptococcus agalactiae*^c^ *(GBS)^S^ (10)*	8	15	<125	<125^(Az)^			32	47^(P)^			(59)
*Staphylococcus aureus (4729)*	60	>4000	>2,000	>2,000^(Er)^	<500	>4,000	1,000	>2,000^(O)^			(67)
*Coagulase-neg Staph. (CoNS) (862)*	60	>4,000	>2,000	>2,000^(Er)^	4,000	>4,000	>2,000	>2,000^(O)^			(67)
*Haemophilus influenzae (150)*	1,000	2,000	1,000	4,000^(Az)^	<500	<500	<1,000	2,000^(AC)^			(67)
*Neisseria gonorrhoeae (246)*	125	250	500	8,000^(Az)^			1,000	16,000^(A)^			(65)
*Chlamydia trachomatis (10)*	250	250	125	125^(Az)^					60	60	(77)
*Mycoplasma pneumoniae (38)*	0.03	0.125	0.25	0.5^(Az)^	500	500			125	250	(74)
*Mycoplasma hominis (13)*	4	8	2,000	4,000^(Az)^	250	500			125	8,000	(74)
*Mycoplasma genitalium (40)*	<1	1,000	8	>8,000^(Az)^					250	1,000	(61)
*Ureaplasma urealyticum*^d^ *(10)*	8	31	2,000	4,000^(Az)^	500	1,000			1,000	16,000	(74)
*Ureaplasma parvum*^d^ *(10)*	8	16	2,000	4,000^(Az)^	500	2,000			8,000	16,000	(74)

*^a^(Er) erythromycin; (Az) azithromycin*.

*^b^(P) penicillin G; (AC) Amoxicillin–clavulanic acid; (O) oxacillin; (A) ampicillin*.

*^c^R, macrolide resistant; S, macrolide susceptible*.

*^d^More recent data from the analysis of 100 strains of Ureaplasma spp. (U. parvum and U. Urealyticum combined) suggest that the solithromycin MIC_90_ is 125 ng/ml and 2000 ng/ml for azithromycin (75)*.

*Solithromycin data are highlighted in the shaded text*.

Solithromycin has been shown to be well tolerated and relatively free of adverse effects, with the most frequent complaint being GI disturbance and nausea that has not been dose-limiting (57, 69, 78, 79). It is not extensively metabolized in humans and is eliminated essentially unchanged via biliary excretion; furthermore, its plasma pharmacokinetics are not altered in patients with mild and moderate renal impairment (80). Two large global Phase 3 trials in community-acquired bacterial pneumonia have recently been completed using both oral and intravenous solithromycin administration (57). The standard oral regimen for solithromycin is 800 mg on day 1 followed by 400 mg daily for 4 days (78). However, a recent clinical trial demonstrated that a single 1000 mg dose of solithromycin eradicates *Neisseria gonorrhoeae* infection at oral, rectal, and genital sites (79).

## Transplacental Pharmacokinetics of Solithromycin: Significance and Implications

**Figure 2:**
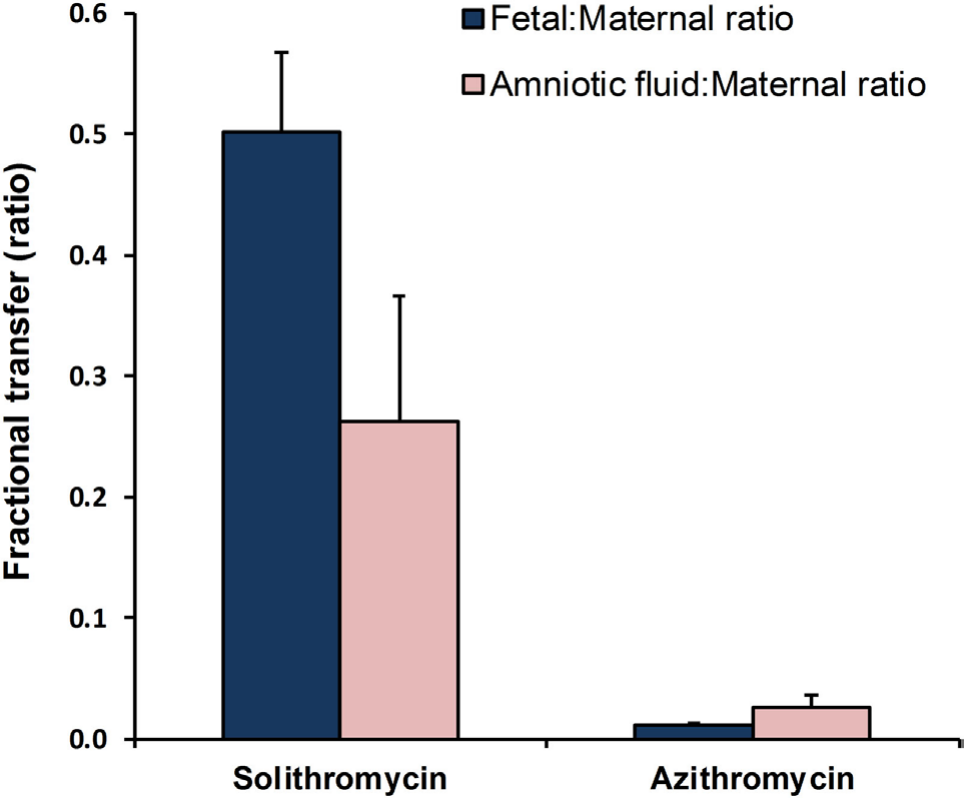
**Comparisons of maternal-to-fetal and maternal-to-amniotic transfer efficiency of solithromycin vs. azithromycin in the pregnant sheep model (10 and 5 mg/kg, respectively)**. Data are mean ± SD; taken from Refs. (55, 82), respectively.

**Figure 3:**
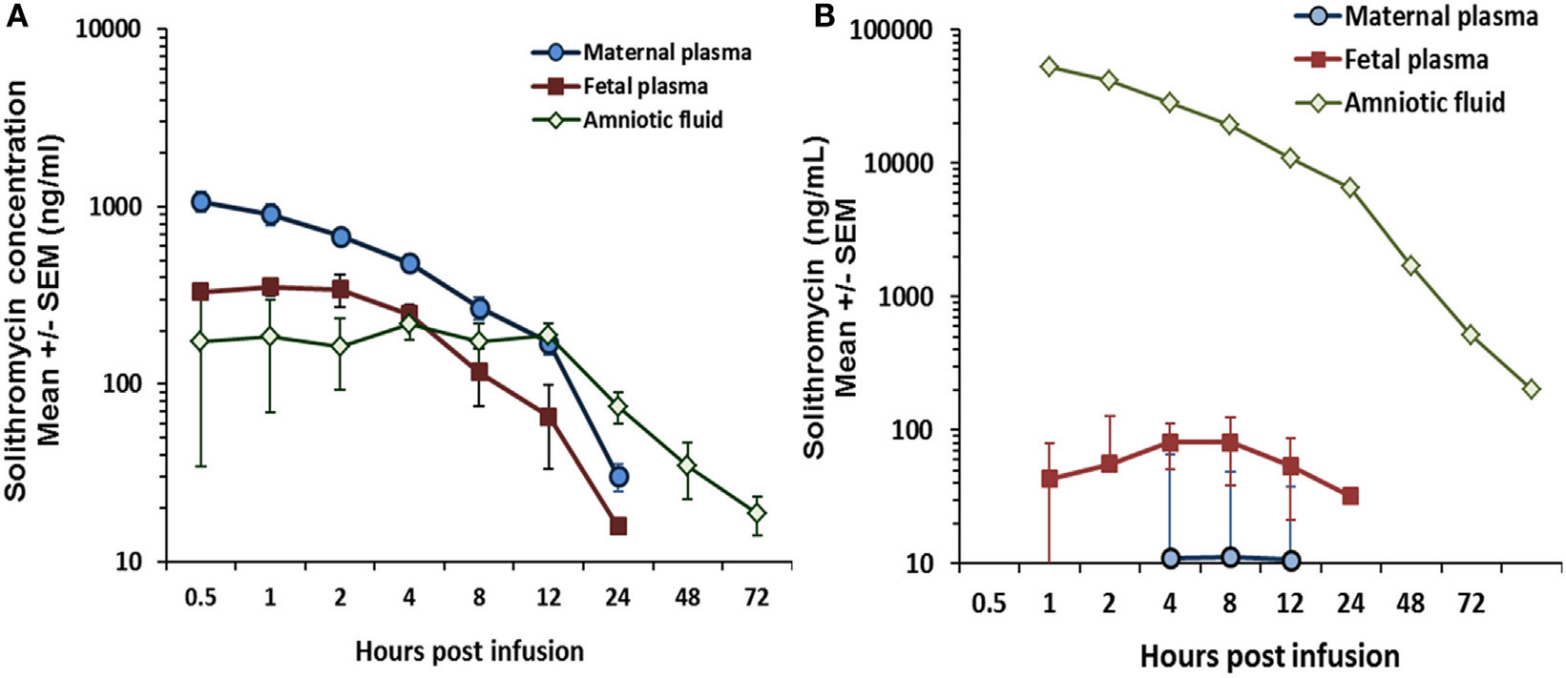
**(A)** Biodistribution of solithromycin in pregnant sheep, showing concentrations in the maternal, fetal, and amniotic fluid (AF) compartments after maternal intravenous administration (10 mg/kg); **(B)** Plasma and AF concentration data in the same model after intra-amniotic injection (1.4 mg/kg fetal weight); azithromycin and solithromycin data taken from Ref (82).

Critically, unlike preexisting macrolides, there is strong evidence that the M:F passage of solithromycin is comparatively efficient. We have shown in an *ex vivo* perfusion model that solithromycin readily crosses the human placenta and reaches effective concentrations in the fetal compartment (81). The M:F transfer ratio is around 0.4–0.6 for the human placenta, while in the pregnant sheep model the M:F transfer ratio of solithromycin was 0.3–0.5 – more than 10-fold greater than erythromycin and azithromycin at similar doses (5–10 mg/kg) (Figure 2) (82). Although ovine and human placentation differ, the similarity in apparent M:F transfer between the species (53) suggests that, in this case, the sheep is a good model to investigate biodistribution of this and other macrolides. Our data would indicate that a daily oral regimen of around 10 mg/kg would maintain effective antimicrobial protection to the fetus, achieving fetal plasma concentrations of several hundred nanogram/milliliter (Figure 3A) (82). Levels are likely to increase further with multiple doses, such that the standard dosing regimen is likely to achieve effective levels in the fetus and intra-amniotically, but this requires experimental confirmation. The key structural features responsible for solithromycin’s dramatically enhanced placental permeability are not known.

Importantly, we have also shown in sheep that significant solithromycin levels in the amniotic cavity (the primary site of infection in this context) are achieved after maternal administration (Figure 3A) (82); repeat daily dosing is likely to achieve even higher concentrations due to delayed clearance from AF, and thereby provide enhanced protection against less sensitive organisms (82). The pharmacokinetic profile in the group receiving an intra-amniotic bolus of solithromycin (Figure 3B) showed that high concentrations were achieved and maintained for over 48 h, with a half-life estimate of 16.5 h. However, it should also be pointed out that this route of administration failed to achieve therapeutic levels in either the maternal or fetal circulation (82), highlighting the need for concurrent maternal administration.

## Reproductive and Developmental Toxicity Aspects of Solithromycin

Macrolides (clarithromycin being the exception due to evidence of teratogenic effects in animal studies) are considered safe to use in pregnancy and have been administered to pregnant women for decades. Two recent, large studies of pregnancies in Canada and Israel (each including more than 100,000 women studied over a 10-year timeframe) reported no evidence of congenital malformations (including cardiac abnormalities) associated with antenatal exposure to erythromycin, azithromycin, or clarithromycin (first-, second-, and third-generation macrolides) (83, 84). Exposure during the third trimester was not associated with increased risk of perinatal mortality, low birth weight, preterm birth, or low apgar scores (83). The Israeli study also found no evidence of increased risk of pyloric stenosis (83), a complication reported in several studies to be associated with perinatal and postnatal exposure of infants to macrolides during the first few weeks of life (85, 86). It is important to note that exposure to macrolides in pregnancy (excluding the peri-partum period) was not associated with a similar increased risk (83, 85). The topic was recently discussed by de Vries and Ludvigsson et al., who pointed out some potential causes of the disparities in the literature (87); the event is rare even if the association is correct (88).

A potential concern around increased risk of congenital heart disease and maternal erythromycin exposure (odds ratio 1.92, 95% CI: 1.37–2.68) was raised in a study by Kallen (89), again using data from the Swedish Birth Register. However, a later study of over 13,000 pregnancies (280 of which were exposed to macrolides) failed to find evidence to support such an association (90). Subsequently, the risks of antenatal macrolide exposure on pyloric stenosis and congenital cardiac malformations were specifically examined in a study of almost 5000 infants born with major congenital defects over a 20-year period by Lin et al. (91). These investigators found no evidence of an association between incidence of these complications and macrolide exposure, regardless of trimester of exposure, including exposure to erythromycin specifically (91). Collectively, these studies support the conclusion that macrolides are safe to administer in pregnancy and any risks to the mother and fetus are extremely low or non-existent.

Nevertheless, if solithromycin is to be administered in pregnancy, it needs an excellent safety profile, particularly in light of its ability to cross the placenta (unlike other macrolides) that could theoretically increase the potential for adverse fetal effects. Studies to date show that its tolerability is high and its side-effects profile is favorable compared to other antibiotics. A phase 2 comparator study in adults showed that at the standard oral dose the most common adverse event was diarrhea (4.7%) followed by flatulence and nausea (1.6% each); no cardiac or neurological side effects were reported and the adverse event rate was significantly lower than in patients taking levofloxacin (78). In a series of phase 1 pharmacokinetic/safety studies, no clinically significant effects were seen, although transient increases in liver enzyme levels were observed in 40% of subjects receiving 600 mg daily over 5 days (68).

Available data suggest that the risks of developmental toxicity and/or teratogenesis with solithromycin are extremely low. The effects of orally administered solithromycin on male and female fertility and early embryonic development to implantation have been evaluated in rats and rabbits (Cempra Inc.: unpublished findings on file). No changes were noted in estrus cycles or sperm parameters in the Segment I study at the maximum dose tested (220 mg/kg). In Segment II studies, some maternal toxicity was observed at the highest dose (decreases in body weight and food consumption and treatment-related clinical signs). The “no observed adverse effect limit” for developmental toxicity was 110–220 mg/kg. No evidence of a teratogenic effect on the fetuses was evident in any treatment group.

Solithromycin has also been evaluated in three *in vitro* genetic toxicology assays and was not mutagenic or clastogenic in any of these assays. In our recent sheep studies, solithromycin-treated animals exhibited no clear evidence of hepatotoxicity (48), although the studies were not designed to specifically address toxicity. Detailed, long-term safety studies have not been carried out in this model.

An emerging concern relating to the use of antibiotics during pregnancy relates to potential adverse effects mediated by perturbation of the maternal–neonatal microbiome by, among other things, antibiotics (92–95). There is now strong evidence that maternal antibiotic exposure can cause neonatal dysbiosis and influence perinatal microbiome development, with potentially significant effects on many developmental processes (96, 97). However, it remains to be seen how dose, timing, and duration of exposure, not to mention the nature of the antibiotic itself, impacts upon these effects. It will be important to assess the effects of maternal solithromycin treatment on both the maternal microbiome (gut, vagina, and other sites) and the infant microbiome and correlate these with developmental outcomes, growth, and the incidence of allergic (98) and metabolic disorders (99, 100). At this point in time, there are no data on solithromycin’s effects on the gut microbiome. However, due to its efficient absorption by the upper GI tract, it is expected that solithromycin’s effects on lower GI tract microflora (typically sampled for microbiome studies) will be markedly less than other macrolides. To address this question, and to assess the short-term and longer-term effects of solithromycin administration on maternal microbiota, a series of studies are planned for the near future.

## Anti-Inflammatory Properties of Solithromycin

It is now widely accepted that fetal and intra-amniotic inflammation, which occurs as a consequence of intrauterine infection or exposure to non-infection inflammatory agents, must be prevented in order to protect the fetus and maximize the benefits of antenatal/perinatal antimicrobial treatment (4, 101, 102). A number of pharmacological strategies have been evaluated by different researchers in order to achieve this effectively and safely. We have focused on the use of cytokine suppressive anti-inflammatory drugs (CSAIDs), in particular agents that block inflammatory signaling via NF-κB and p38MAPK pathways; an overview of these studies is presented in a companion article in this series (101). A key issue of such approaches, however, is the mode of delivery and the prevention of side effects. When given maternally, the dose administered must be large enough to ensure that the drug achieves effective anti-inflammatory concentrations in the amniotic cavity, but not sufficiently high to cause maternal toxicity or off-target side effects. To address this problem, and also overcome the lack of permeability of some agents across the human placenta, we have investigated intra-amniotic delivery of agents. We have been able to demonstrate benefits of this approach with several agents in animal and *ex vivo* models (103–105). However, while this mode of delivery has some clear benefits, including the ability to achieve quite high drug concentrations in the amniotic cavity without risk of significant maternal or fetal exposure (where fetal drug uptake is low), it also has drawbacks in that any maternal inflammation remains untreated. This limitation may represent a lost opportunity to improve pregnancy outcomes in a sub-group of women.

In this context, solithromycin may provide additional pharmacological benefits as it is also an effective anti-­inflammatory agent. As it would be given maternally, and crosses the placenta relatively efficiently, solithromycin therapy may be able to achieve the benefits of inhibiting both maternal and intrauterine inflammation in addition to its antimicrobial actions – depending upon the dose administered. In a key publication, Kobayashi et al. reported that solithromycin exhibits significant NF-κB-mediated anti-inflammatory effects (reduced cytokine and matrix-metalloproteinase (MMP)-9 expression) in human monocytes and peripheral blood mononuclear cells at concentrations ~10–40 μM (106, 107). Importantly, the anti-inflammatory effects were ≥10-fold more potent than erythromycin, clarithromycin, or azithromycin. The structural features responsible for the anti-inflammatory properties of macrolides have not been identified, although the macrocyclic ring is likely to be crucial (106); the specific structural characteristics responsible for solithromycin’s enhanced anti-inflammatory properties are unknown. *In vivo* suppression of neutrophilia and MMP-9 activity in a mouse model was also achieved with solithromycin treatment (100 mg/kg) after exposure to a non-infectious inflammatory stimulus (107). The mechanism of action appears to be, in part, a combination of effects on HDAC2 activity (enhanced) and Akt phosphorylation (inhibited), via increased protein phosphatase PP2A activity (106). Solithromycin also has significant effects on NF-κB activity, probably mediated through enhanced dissociation of IκBα from p65/RelA, as has been demonstrated for other macrolides (107–110). These findings have stimulated interest in the use of solithromycin administration for treatment of chronic obstructive lung disease, asthma, and non-alcoholic steatohepatitis, with a series of investigational studies now underway.

In an *in vitro* study of human placental tissues, we confirmed the anti-inflammatory properties of solithromycin in human placentas, reporting inhibition of pro-inflammatory cytokine production by the antibiotic; however, this effect was only observed at relatively high concentrations (≥33 μg/ml, or ~40 μM) at which a decline in cell viability was observed in this model (81). Effects of a similar magnitude and potency were also observed in (maternal) decidual cells. Furthermore, data from the pregnant sheep model also support an anti-inflammatory effect of solithromycin in pregnancy. We previously reported that solithromycin, delivered maternally (10 mg/kg i.v.), decreases the levels of mRNA expression of IL-1beta, IL-6, IL-8, and *MCP2* in fetal skin of *Ureaplasma parvum*-exposed animals (48). No significant effects on the inflammation scoring of lung or chorioamnion were observed in this study, although some non-significant trends were observed toward reductions in lung cytokine expression, inflammatory histology, and cord blood white blood cell count (48).

These findings raise questions as to whether a sufficiently high dose of the antibiotic can be given to achieve anti-inflammatory benefits without toxicity. Our studies, assuming that they can be extrapolated to the pregnant woman, would suggest that large amounts of solithromycin would need to be given maternally to suppress placental and intra-amniotic inflammation, running the risk of placental toxicity and possibly other adverse effects. Administration via the intra-amniotic route by ultrasound-guided injection would be able to achieve the levels necessary to exert significant anti-inflammatory effects within the amniotic cavity without maternal or placental exposure. This may be an advantageous strategy in some clinical situations, in which the fetus is at risk and particularly rapid antimicrobial and anti-inflammatory therapy is required. Appropriate randomized clinical trials in pregnancy would be required to ensure that the benefits outweigh the potential risks of the intervention.

## Conclusions, Applications, and Future Research Directions

We believe solithromycin has exciting potential for the treatment of intrauterine infections, prevention of preterm birth, and also treatment of perinatal and postnatal infections. Its pharmacodynamic and pharmacokinetic properties are ideally suited to these applications, and our data on transplacental passage and AF accumulation suggest that this antibiotic may represent a major advance in antimicrobial therapy in pregnancy.

There are three main obstetric scenarios where solithromycin therapy may be particularly beneficial. The first is in the prophylactic treatment of asymptomatic women at high risk of preterm birth in the first half of pregnancy. The strategy requires the ability to identify women who are at risk of intrauterine infection and, thus, target them for solithromycin treatment (35). Prognostic indications, in addition to standard clinical risk factors, could be abnormal vaginal microbiota or presence of particular microbial profiles or species (15, 16, 111–113), or a short cervix with evidence of inflammatory changes (114–116). The second situation is in women with PPROM. In these pregnancies, macrolides have been shown to have significant benefits in terms of neonatal outcomes (117); with its far superior efficacy profile and ability to treat the fetus *in utero*, it is likely that solithromycin would be much more beneficial than erythromycin for this indication. Finally, solithromycin may be effective in improving neonatal outcomes in women presenting with preterm labor and intact membranes, providing both antimicrobial and anti-inflammatory benefits to the fetus prior to delivery. In all of these scenarios, co-administration with a more potent anti-inflammatory agent may further improve outcomes. Clinical trials to explore all of these applications are warranted, once pharmacokinetic studies have been conducted to establish safe and effective dosing regimens in early, mid, and late pregnancy. Assessment of the short- and long-term effects of antenatal solithromycin therapy on the vaginal, gastrointestinal, and neonatal microbiomes would also be warranted prior to trials of its therapeutic effectiveness in pregnancy.

## Author Contributions

JK conceptualized the article and prepared the first draft, including the figures and tables. MP, MK, DI, and JN contributed to various sections of the text and provided input, comment, and changes to the entire manuscript.

## Conflict of Interest Statement

Dr. Prabha Fernandes of Cempra Inc. (Chapel Hill, NC, USA) supplied the solithromycin used in the pertinent studies included in this review, and provided comments on the manuscript, but had no involvement in the design of the studies or the analysis of the data. The authors declare that the research was conducted in the absence of any commercial or financial relationships that could be construed as a potential conflict of interest.
